# Transcriptional coupling (Mfd) and DNA damage scanning (DisA) coordinate excision repair events for efficient *Bacillus subtilis* spore outgrowth

**DOI:** 10.1002/mbo3.593

**Published:** 2018-03-13

**Authors:** Luz I. Valenzuela‐García, Víctor M. Ayala‐García, Ana G. Regalado‐García, Peter Setlow, Mario Pedraza‐Reyes

**Affiliations:** ^1^ Department of Biology University of Guanajuato Guanajuato Mexico; ^2^ Department of Molecular Biology and Biophysics UConn Health Farmington CT USA

**Keywords:** *Bacillus subtilis*, DisA, germination/outgrowth, NER, TCR

## Abstract

The absence of base excision repair (BER) proteins involved in processing ROS‐promoted genetic insults activates a DNA damage scanning (DisA)‐dependent checkpoint event in outgrowing *Bacillus subtilis* spores. Here, we report that genetic disabling of transcription‐coupled repair (TCR) or nucleotide excision repair (NER) pathways severely affected outgrowth of Δ*disA* spores, and much more so than the effects of these mutations on log phase growth. This defect delayed the first division of spore′s nucleoid suggesting that unrepaired lesions affected transcription and/or replication during outgrowth. Accordingly, return to life of spores deficient in DisA/Mfd or DisA/UvrA was severely affected by a ROS‐inducer or a replication blocking agent, hydrogen peroxide and 4‐nitroquinoline‐oxide, respectively. Mutation frequencies to rifampin resistance (Rif^r^) revealed that DisA allowed faithful NER‐dependent DNA repair but activated error‐prone repair in TCR‐deficient outgrowing spores. Sequencing analysis of *rpoB* from spontaneous Rif^r^ colonies revealed that mutations resulting from base deamination predominated in outgrowing wild‐type spores. Interestingly, a wide range of base substitutions promoted by oxidized DNA bases were detected in Δ*disA* and Δ*mfd* outgrown spores. Overall, our results suggest that Mfd and DisA coordinate excision repair events in spore outgrowth to eliminate DNA lesions that interfere with replication and transcription during this developmental period.

## 1 INTRODUCTION

*Bacillus subtilis* spores are metabolically dormant as well as resistant to a number of DNA‐damaging agents, including, heat, radiation, desiccation, extreme pH, and oxidizing agents (Setlow, 2007). This DNA resistance is due in large part to a group of DNA‐binding, acid‐soluble spore proteins (α/β‐SASPs), synthesized during the last stages of sporulation (Setlow, 1988). After detecting appropriate conditions, spores can return to vegetative growth through a two‐step process termed germination and then outgrowth (Setlow, 2003; Setlow, Wang, & Li, 2017). This process is triggered by specific germinants, generally amino acids or sugars that are specifically sensed by receptors in the spore's inner membrane (Paidhungat & Setlow, 2002; Setlow, 2003). This receptor‐germinant interaction activates several events, including release of dipicolinic acid and monovalent and divalent cations from the spore core, hydrolysis of the spore cortex peptidoglycan, and uptake of water into the spore core to levels comparable to those in growing cells. The full hydration of the spore core completes germination and allows resumption of normal enzyme activity in the spore core including the replication and transcription machineries necessary for initiation of spore outgrowth (Setlow, 2003). During outgrowth, the α/β type SASPs are degraded, freeing spore DNA for transcription, and eventually for replication (Setlow, 1988, 2007). Results from a transcriptomic study revealed that genes encoding repair proteins are expressed during early (5–25 min) and late (40–50 min) outgrowth stages (Keijser et al., 2007). Furthermore, the free amino acids produced from proteolysis of α/β‐SASPs support much of the metabolism early in spore outgrowth (Setlow, 2007).

The ability of spores to germinate and propagate depends on their genomic integrity (Setlow & Setlow, 1996). DNA repair cannot take place in metabolically dormant spores, therefore, DNA lesions generated by chemical or physical factors accumulated during the variable periods of spore dormancy, need to be repaired during spores’ return to vegetative growth (Nicholson, Munakata, Horneck, Melosh, & Setlow, 2000; Setlow, 2007). It has been proposed that DNA lesions accumulated in dormant spores must be eliminated soon after completion of germination by DNA repair proteins produced and stored in the spore during its development (Pedraza‐Reyes, Ramírez‐Ramírez, Vidales‐Rodríguez, & Robleto, 2012).

In *B. subtilis*, the entrance into sporulation and outgrowth is modulated by DNA damage and conditions that interfere with chromosomal replication (Bejerano‐Sagie et al., 2006; Burkholder, Kurtser, & Grossman, 2001; Campos et al., 2014; Rahn‐Lee, Gorbatyuk, Skovgaard, & Losick, 2009). Thus, DisA, a DNA‐binding protein, scans the chromosome during sporulation and if DNA damage is detected, spore formation is blocked before the asymmetric sporulation division (Bejerano‐Sagie et al., 2006). Structural and biochemical studies have shown that DisA forms an octamer and also synthesizes the small molecule cyclic diadenosine monophosphate (c‐di‐AMP) (Witte, Hartung, Büttner, & Hopfner, 2008). DisA's diadenylate cyclase activity when bound to DNA is moderately reduced by 3′‐ and 5′‐ DNA flaps and is strongly suppressed on branched nucleic acids such as Holliday junctions or stalled replication forks (Witte et al., 2008). DisA and c‐di‐AMP have been associated with DNA integrity during sporulation and vegetative growth in *B. subtilis* (Gándara & Alonso, 2015; Oppenheimer‐Shaanan, Wexselblatt, Katzhendler, Yavin, & Ben‐Yehuda, 2011).

As noted above, the return to vegetative growth of *B. subtilis* spores represents a stage with increased oxidative stress due to the full hydration of the spore core during germination and activation of metabolism during outgrowth (Campos et al., 2014; Ibarra et al., 2008). The oxidative damage inflicted on DNA by reactive oxygen species (ROS) can be counteracted by KatX and the apurinic, apyrimidinic (AP) endonucleases Nfo, and ExoA (Bagyan, Casillas‐Martinez, & Setlow, 1998; Ibarra et al., 2008). In the absence of Nfo and ExoA, DisA delays chromosomal replication and spore outgrowth until the genome is free of damage. Of note, DisA is not packaged into the forespore compartment during sporulation, but is synthesized during outgrowth (Campos et al., 2014).

During outgrowth, full reconstitution of many metabolic pathways, as well as nutrient uptake and cell replication requires macromolecular synthesis, which can be launched upon production of ATP (Keijser et al., 2007). In outgrowing spores, protein synthesis is dependent on de novo transcription; therefore, expression of a number of genes is required during the first minutes of outgrowth (Setlow & Primus, 1975; Setlow, 1975). In growing bacteria, genetic lesions occurring in transcriptionally active genes are preferentially repaired, in particular those occurring in the template strand, in a process termed transcription‐coupled repair (TCR) (Hanawalt & Spivak, 2008; Selby, Witkin, & Sancar, 1991). In this process, the mutation frequency decline protein (Mfd) responds to RNA polymerase stalled by bulky or noncoding lesions and recruits the nucleotide excision repair system (NER) to the lesions through Mfd's interaction with the UvrA protein (Hanawalt & Spivak, 2008; Selby & Sancar, 1993). In *B. subtilis* the role of Mfd in vegetative growth and stationary phase has been well characterized (Ayora, Rojo, Ogasawara, Nakai, & Alonso, 1996; Pybus et al., 2010; Ross et al., 2006; Zalieckas, Wray, Ferson, & Fisher, 1998). Recent experimental evidence has further revealed that *mfd* is expressed in the forespore compartment of the sporulating cell and that TCR is required to contend with the noxious effects of bulky DNA lesions during spore morphogenesis (Ramírez‐Guadiana et al., 2013). It was also proposed that Mfd stored in spores could play a role in processing DNA damage during spore outgrowth (Ramírez‐Guadiana et al., 2013).

The efficient return of spores to vegetative growth requires a chromosome free of damage for appropriate transcription and replication (Keijser et al., 2007; Setlow & Setlow, 1996). Therefore, spore outgrowth offers the opportunity to study how Mfd and DisA modulate excision repair events to ensure efficient spore outgrowth. Experimental evidence presented in this study shows that Mfd, DisA, and the NER protein UvrA coordinate excision repair events to deal with genetic lesions that interfere with transcriptional and replication events in outgrowing *B. subtilis* spores.

## 2 MATERIALS AND METHODS

### 2.1 Strain construction and culture conditions

The *B. subtilis* strains used in this study were derived from strain PS832, a prototrophic derivative of strain 168 and are listed in Table S1. The strains were constructed using standard molecular biology techniques (Sambrook & Russell, 2001).

Competent cells of *B. subtilis ∆disA* (PERM733) (Campos et al., 2014) were transformed with chromosomal DNA of *B. subtilis* strains ∆*mfd* (PERM938) (Ramírez‐Guadiana et al., 2013) and ∆*uvrA* (PERM985) (Ramírez‐Guadiana et al., 2012), generating strains ∆*disA mfd* (PERM1333) and ∆*disA uvrA* (PERM1342), respectively. Chromosomal DNA from ∆*mfd* (PERM938) and ∆*uvrA* (PERM985) strains were used to transform competent cells of *B. subtilis* PS832, thus generating strains ∆*mfd* (PERM1460) and ∆*uvrA* (PERM1461), respectively.

A gene construct to disrupt *disA* was generated as follows. A 307‐bp DNA fragment extending from nucleotides (nt) 275–582 from the *disA* open reading frame (ORF) was released from plasmid pPERM732 (Campos et al., 2014) by digestion with the enzymes *Eco*RI and *Bam*HI and cloned into the pMutin4cat vector (Barajas‐Ornelas et al., 2014). The resulting plasmid pPERM1372 was used to transform competent cells of strain PS832 giving strain *∆disA* (PERM1504). Competent cells of *B. subtilis* PERM1504 were transformed with chromosomal DNA of strains ∆*mfd yqjH* (PERM939) and ∆*mfd yqjW* (PERM940) (Ramírez‐Guadiana et al., 2013) to generate strains *∆disA mfd yqjH* (PERM1510) and *∆disA mfd yqjW* (PERM1511), respectively. The appropriate recombination events into the homologous loci were confirmed by PCR using specific oligonucleotide primers (data not shown).

Liquid cultures of *B. subtilis* were grown routinely in Luria‐Bertani (LB) medium (Miller, 1972). When required, erythromycin (Er; 5 μg ml^−1^), chloramphenicol (Cm; 5 μg ml^−1^), kanamycin (Kn; 10 μg ml^−1^), tetracycline (Tet; 10 μg ml^−1^), or rifampicin (Rif; 10 μg ml^−1^) were added to media. *E. coli* cultures were grown in LB medium supplemented with Cm (25 μg ml^−1^) or ampicillin (Amp; 100 μg ml^−1^). Solid media were obtained by adding bacteriology grade agar (15 g L^−1^) to the liquid media. Liquid cultures were incubated at 37°C with vigorous aeration. Cultures on solid media were incubated at 37°C in the dark.

Spores of all strains were prepared at 37°C on Difco sporulation medium (DSM) (Schaeffer, Millet, & Aubert, 1965) agar plates without antibiotics, harvested and purified by water washing and stored as described previously (Nicholson & Setlow, 1990). All dormant spore preparations used in this work were free (≥98%) of growing cells, germinated spores, and cells debris, as determined by phase‐contrast microscopy. Spores were generally used at an optical density at 600 nm (OD_600_) of 0.5 corresponding to 0.75 × 10^8^ viable spores/ml.

### 2.2 Spore germination and outgrowth

Spore germination and outgrowth were performed in 2 × Schaeffer′s glucose (2 × SG) liquid medium (Schaeffer et al., 1965) supplemented with 10 mmol L^−1^ L‐alanine. Spores in water were first heat shocked for 30 min at 70°C, cooled on ice, and inoculated into germination medium at 37°C to obtain an initial OD_600_ of ~0.5. Where indicated, 0.5 mmol L^−1^ hydrogen peroxide (H_2_O_2_) (Sigma‐Aldrich, St. Louis MO) or 2 μmol L^−1^ 4‐NQO (4‐Nitroquinoline‐1‐Oxide) (Sigma‐Aldrich, St. Louis MO), equivalent to a 30% lethal dose of each drug, were added to cultures after most spore germination had taken place; that is, ~15 min after the mixing of spores with germinants. The OD_600_ of cultures were monitored with an Ultrospec 2000 spectrophotometer (Pharmacia, Manassas Park, VA), and the values were plotted as a fraction of the initial OD_600_ (OD_600_ at time *t*/initial OD_600_) versus time. The rates of germination of *disA mfd*,* disA uvrA,* and wild‐type spores were determined in 25 mmol L^−1^ Tris‐HCl (pH 7.4) plus L‐alanine of spore cultures. To this end, the fall in the relative OD_600_ values was monitored over a period of 30 min and the linear portion employed to calculate the slope. The rate of germination of wild‐type spores was refereed as 100%.

### 2.3 Determination of chromosomal DNA content

To quantify genomic DNA from spores germinated and outgrown, chromosomal DNA was isolated as follows. Aliquots (3 ml; 1.5 × 10^8^ viable spores/ml) of WT, *∆disA mfd* and ∆*disA uvrA* dormant spores that had germinated for 30, 60, 90, and 120 min in 2 × SG were collected by centrifugation (13,500*g* for 2 min). The pellet of cells was washed two times with 1 ml of lysis buffer (50 mmol L^−1^ EDTA, 100 mmol L^−1^ NaCl [pH 7.5]), suspended in 0.3 ml of the same buffer and subsequently processed to isolate the RNA‐free chromosomal DNA from the fraction that was directly susceptible to lysozyme degradation as previously described (Campos et al., 2014). The fraction of lysozyme‐resistant cells was pelleted by centrifugation. This pellet, which consisted of lysozyme‐resistant spores likely containing intact spore coats, was subjected to spore coat removal (Nicholson & Setlow, 1990), washed five times with STE buffer (150 mmol L^−1^ NaCl, 10 mmol L^−1^ Tris‐HCl [pH 8], 10 mmol L^−1^ EDTA), and processed for chromosomal DNA isolation (Campos et al., 2014). After RNAse treatment, the chromosomal DNA isolated from both fractions was quantified by UV spectrophotometry (Sambrook & Russell, 2001). The DNA values from both fractions were combined to obtain the total DNA content.

### 2.4 Microscopy analysis

Cell morphology and chromosome structure during spore germination/outgrowth were analyzed by fluorescence microscopy. Cell samples (1 ml) collected at different times during spore germination/outgrowth, were centrifuged (11,500*g*, 2 min) and mixed with 0.2 ml of fixative solution (3% (v/v) paraformaldehyde and 5% (v/v) glutaraldehyde in HEPES‐buffered saline [273 mmol L^−1^ NaCl, 9.9 mmol L^−1^ KCl, 1.27 mmol L^−1^ Na_2_HPO_4_.2H_2_O, 11.1 mmol L^−1^ dextrose, 42 mmol L^−1^ HEPES (pH 7)]). After 30 min at room temperature, fixation was continued on ice for 50 min. The samples were washed twice by centrifugation with PBS and suspended in 100 μl of GTE [5 mmol L^−1^ glucose, 25 mmol L^−1^ Tris‐HCl, 10 mmol L^−1^ EDTA (pH 8)]. Aliquots (10 μl) of this cell suspension were supplemented with 2 μg ml^−1^ of 4′, 6′‐diamidino‐2‐phenylindole (DAPI) to stain the chromosome and with FM4‐64 (5 μg ml^−1^) to stain cell membranes and stained samples kept at room temperature for 15 min. For microscopy, cell samples were prepared as previously described (Ayala‐García, Valenzuela‐García, Setlow, & Pedraza‐Reyes, 2016). Fluorescence microscopy was performed with a ZEISS Axioscope A1 microscope equipped with an AxioCam ICc1 camera. Images were acquired with the AxioVision V 4.8.2 software and adjusted only for brightness and contrast. Exposure times were typically 0.2 s for DAPI and 0.5 s for FM4‐64. Excitation and emission wavelengths employed were 350 and 470 nm for DAPI, and 506 and 750 nm for FM4‐64, respectively.

### 2.5 Analysis of spontaneous and induced mutation frequencies

Determination of spontaneous and H_2_O_2_ or 4‐NQO induced mutation frequencies to rifampicin resistance (Rif^r^) was performed as follows. Spores were adjusted to a final OD_600_ of 0.5 in 2× SG medium, supplemented with 10 mmol L^−1^ of L‐alanine and treated (induced) or not (spontaneous) with the DNA‐damaging agents, H_2_O_2_ (0.5 mmol L^−1^) or 4‐NQO (2 μmol L^−1^), which were added to cultures 15 min after the initiation of germination. 10 ml of cell samples collected 180 min after initiation of germination, were washed with 10 ml of phosphate‐buffered saline (PBS; 0.7% Na_2_HPO_4_, 0.3% KH_2_PO_4_, 0.4% NaCl [pH 7.5]) and resuspended in 1 ml of the same buffer. Aliquots of cells were plated on six LB medium plates containing 10 μg ml^−1^ of rifampicin, and Rif^r^ colonies were counted after 2 days of incubation at 37°C. The number of cells used to calculate the frequency of mutation to Rif^r^ was determined by plating aliquots of appropriate dilutions on LB medium plates without rifampicin and incubating the plates for 24 h at 37°C.

### 2.6 Identification of spontaneous rpoB mutations conferring rifampin resistance

Rif^r^ colonies spontaneously generated from outgrown spores of the strains of interest were randomly chosen, resuspended in 100 μl of nuclease free‐water and subject to cell lysis by heating the cell suspension at 95°C for 5 min (Nicholson & Maughan, 2002). The cell lysates were employed to PCR amplify a 716‐bp fragment from *rpoB* with Vent DNA polymerase (New England Bio‐Labs, Ipswich, MA) and the oligonucleotide primer set, RpoBFW 5′‐CGTCCTGTTATTGCGTCC‐3 (forward) and RpoBRV 5′‐GGCTTCTACGCGTTCAACG‐3′ (reverse). The amplified 716‐bp *rpoB* product contained the three hot‐spot clusters (nt +1353 to +2069 relative to the ORF of the *rpoB* gene) where mutations confer Rif^r^ in many bacteria including *B. subtilis* (Campbell et al., 2001). The amplified *rpoB* products were subjected to DNA sequencing to identify specific mutations conferring rifampicin resistance. The sequencing was performed in both directions of the *rpoB* PCR product of 20 clones from wild‐type, ∆*disA* strain, ∆*mfd s*train, and ∆*disA mfd* strains. Sequencing was carried out by Functional Biosciences, Inc. (Madison, WI) and the Genomic Services Unit in Langebio, Cinvestav, México.

### 2.7 Monitoring of the SOS response in outgrowing spores of *B. subtilis* wild‐type, *∆disA*,* ∆mfd*,* ∆uvrA*,* ∆disA/uvrA,* and *∆disA/mfd* strains

To investigate if the lack of Mfd and UvrA in the Δ*disA* background induces the SOS response during the return to vegetative growth of *B. subtilis* spores, we introduced a *recA‐gfpmut3a* fusion (Ramírez‐Guadiana, Barajas‐Ornelas, Corona‐Bautista, Setlow, & Pedraza‐Reyes, 2016) into the *amyE* locus of the wild‐type, *∆disA*,* ∆uvrA*, ∆*mfd,* ∆*disA/uvrA,* and ∆*disA mfd* strains, generating strains PERM1549, PERM1550, PERM1548, PERM1561,PERM1559, and PERM1560, respectively (Table S1). Wild‐type, *∆disA, ∆uvrA*, ∆*disA/uvrA, ∆mfd,* and ∆*disA/mfd* strains carrying the empty vector pDR111 at the *amyE* locus were also generated (Table S1) to quantify the basal fluorescence emitted by cells with these genetic backgrounds. Spores of the different strains carrying the *recA‐gfpmut3a* fusion or the pDR111 empty vector were obtained and purified as described above. Heat shocked spores were inoculated into 2 × SG medium supplemented with 10 mmol L^−1^ L‐alanine to an OD_600 nm _= 0.5 and the cultures were shaken at 37°C/250 rpm. After 15 min, each culture was splitted in two equal subcultures; one subculture was made 250 ng/ml in Mitomycin‐C (M‐C) and the other was left untreated. After 60 min of incubation at 37°C with shaking, samples of 3 ml were collected from both subcultures, pelleted by centrifugation (10,000*g* for 2 min), washed two times with PBS and resuspended in 1 ml of the same buffer. Decimal serial dilutions were prepared from untreated an M‐C‐treated cell suspensions in PBS and plated on solid LB medium to determine viable counts. The fluorescence emitted by each cell sample was quantified with a LS55 Perkin Elmer fluorescence spectrometer (PerkinElmer, Waltham, MA) set at excitation and emission wavelengths of 498 and 512 nm, respectively. The basal values of fluorescence emitted by cell samples of cultures prepared with strains carrying the empty vector pDR111 (Table S1), and with or without M‐C, were subtracted from the values obtained with the strains harboring the *recA‐gfpmut3a* fusion. The basal values of fluorescence were never superior to 10% (for the noninduced) or 1.5% (for the induced) condition in reference to the strains carrying *recA‐gfpmut3a*.

### 2.8 Statistical analyses

Differences in mutagenesis between untreated and treated with the DNA damage agents H_2_O_2_ or 4‐NQO as well as differences in fluorescence between untreated and treated with M‐C strains were calculated by Mann–Whitney *U* test, and analyses were done using Minitab 17 software. *p *< .05 were considered significant.

## 3 RESULTS

### 3.1 The lack of Mfd or UvrA delays outgrowth of spores lacking DisA

**Figure 1:**
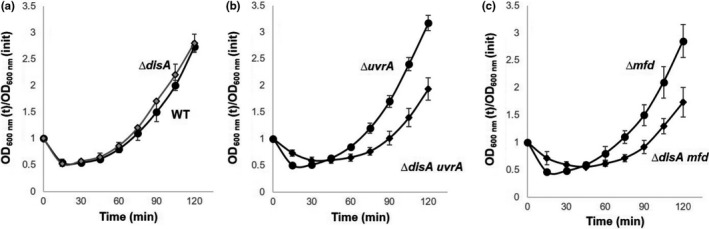
(a–c) Germination and outgrowth of spores of different *B. subtilis* strains. Dormant spores of the wild‐type (●), ∆*disA* (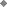), ∆*uvrA* (●), ∆*disA uvrA* (◆), ∆*mfd* (●), and ∆*disA mfd* (◆) strains, were induced to germinate and spore germination and outgrowth were measured by monitoring the OD_600_ of the cultures, all as described in Materials and Methods. Values are averages ± standard deviations for triplicate determinations with different lots of spores; WT, wild type

Oxidative DNA damage is a challenge faced by spores during the return to vegetative growth, as ROS‐promoted lesions, including oxidized bases, AP sites, and single‐strand breaks can be impediments to the transcription and replication machinery during spore outgrowth (Campos et al., 2014; Ibarra et al., 2008). During sporulation, significant amounts of the DNA repair proteins Mfd and UvrA are expressed and packaged in the developing forespore (Ramírez‐Guadiana et al., 2013); in contrast, *disA* is not packaged into the forespore compartment but is synthesized very early in spore outgrowth (Campos et al., 2014). Of note, spores lacking Mfd, UvrA, or DisA alone exhibited germination/outgrowth curves that were indistinguishable from that of wild‐type spores suggesting these proteins have either no or redundant functions in this developmental stage (Figure 1). In support of a redundant function for these proteins, loss of Mfd or UvrA in a strain also lacking DisA generated spores that were delayed significantly in their return to vegetative growth in comparison with spores of the wild‐type strain. Some of this latter delay was due to a slightly slower germination of the *mfd disA* and *uvrA disA* spores compared to that of wild‐type spores (Figure 1). Thus, when germination of *mfd disA*,* uvrA disA,* and wild‐type spores was monitored by the fall in the OD_600_ in 25 mmol L^−1^ Tris‐HCl (pH 7.4) plus L‐alanine of spore cultures (Campos et al., 2014), the rates of germinations of the double mutants was ~90% to that of the wild‐type spores. This small difference was seen with at least two different preparations of these spores. Experimental evidence has revealed that RecA, UvrA, and Mfd‐dependent DNA repair is a relevant process for efficient spore morphogenesis (Ramírez‐Guadiana et al., 2013, 2016). Therefore, some genetic defect resulting from the loss of these repair function seems to generate dormant spores slightly affected in spore germination but bearing much more significant deficiencies in spore outgrowth. Importantly, the *disA uvrA* and *disA mfd* strains exhibited essentially similar doubling times as the wild‐type strain in vegetative growth—that is, 35 ± 2.5, 36 ± 1.2, and 34 ± 2, respectively. Therefore, the strong slow outgrowth exhibited by spores of these mutant strains cannot be attributed to vegetative growth defects.

### 3.2 The delay in outgrowth of *∆disA*/*uvrA* and *∆disA/mfd* spores is accompanied by retardation of chromosome replication

**Figure 2:**
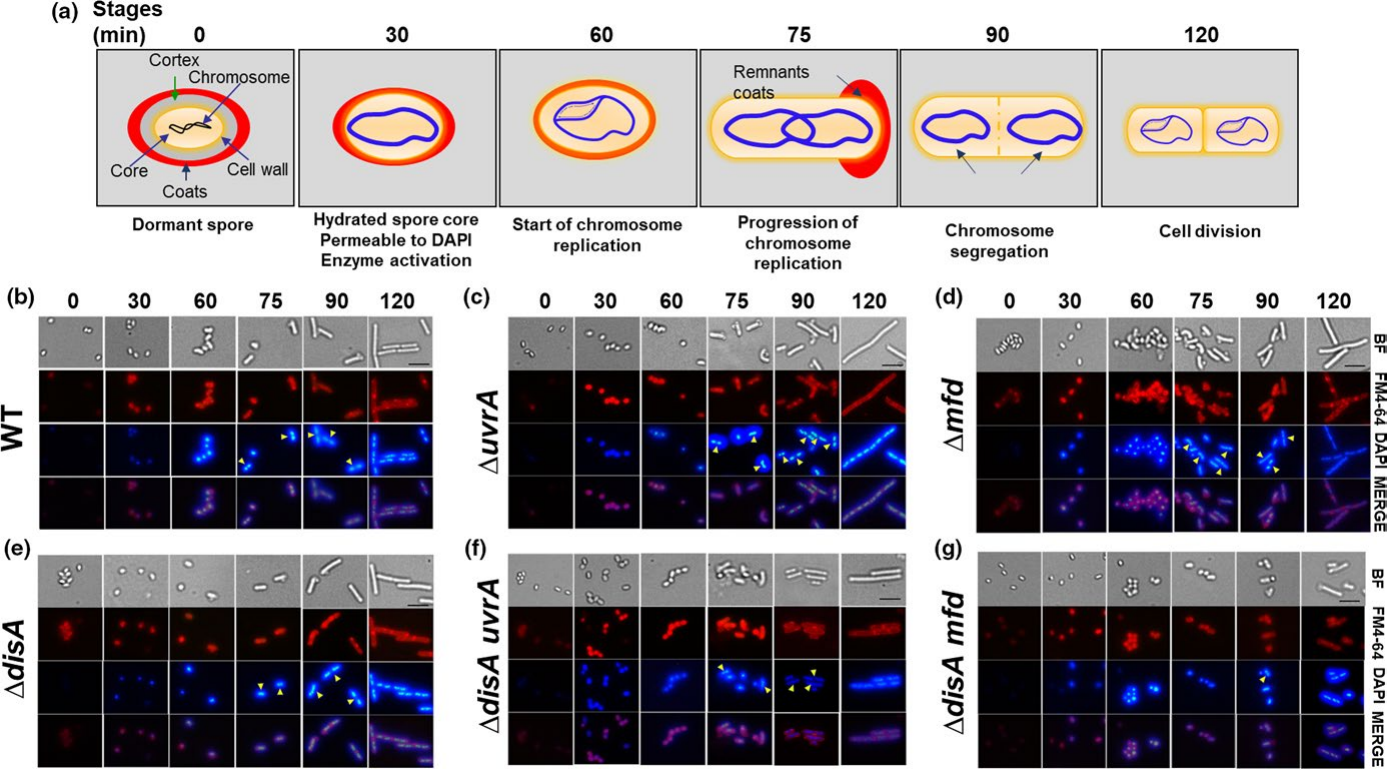
Microscopic analysis of chromosome replication in outgrowing spores of different *B. subtilis* strains. (a) Schematic representation of spore germination/outgrowth stages employed for microscopic analysis. See text for details. (b–g) Dormant spores of wild‐type (b), ∆*uvrA* (c), ∆*mfd* (d), ∆*disA* (e), ∆*disA uvrA* (f), and ∆*disA mfd* (g) strains, were heat shocked and germinated as described in Materials and Methods. At different times (0, 30, 60, 75, 90, and 120 min) during germination/outgrowth, cells were collected, fixed and analyzed by bright‐field (BF) and fluorescence (DAPI and FM4‐64 staining) microscopy as described in Materials and Methods. Overlain images of DAPI and FM4‐64 at each time point are depicted as MERGE. Scale bar, 5 μm. Yellow arrowheads show cells that have replicated (at 75 min) and segregated their chromosome (at 90 min). For each strain, >200 cells were analyzed in at least six different fields

**Figure 3:**
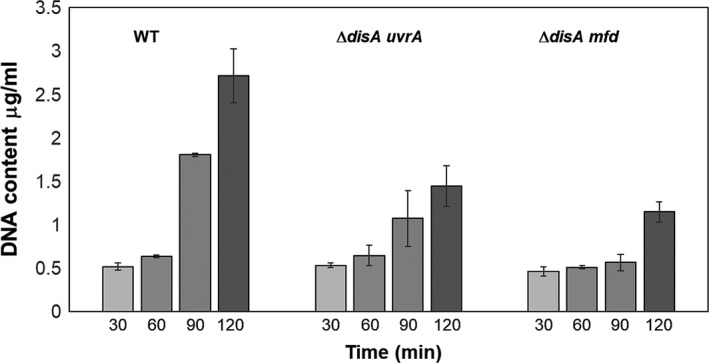
DNA concentration in outgrowing spores of different strains. Levels of DNA in samples from cultures of wild‐type, ∆*disA uvrA* and ∆*disA mfd* spores collected at different times after the germination onset were determined as described in Materials and Methods. The results are the average of duplicate independent determinations with different lots of spores ± SD

The outgrowth defect exhibited by DisA/Mfd‐ and DisA/UvrA‐deficient spores was further examined by epifluorescence microscopy. To this end, spores of these strains as well as spores of the wild‐type strain and those bearing single mutations in *disA*,* mfd,* or *uvrA* were induced to germinate in a medium that supported outgrowth and vegetative cell growth, and samples were collected at different stages during germination/outgrowth, and DNA and membrane were stained with DAPI and FM4‐64 dyes, respectively (Figure 2). With wild‐type spores (Figure 2b), the time points analyzed from 30 to 90 min under the germination conditions employed in this work, corresponded to the physiological state previously defined as “outgrowth” when the germinated spore becomes a growing cell, generally after the first cell division (Setlow, 2003). The following characteristics define the time points chosen for microscopic analysis of wild‐type spores (depicted in Figure 2a,b): (1) 30 min—when the swollen spore has undergone hydrolysis of the cortex peptidoglycan as evidenced by the ability of DAPI to penetrate into the spore core and stain spore DNA; at this time, the spore core is fully hydrated, core proteins have recovered mobility and core enzymes are activated (Setlow, 2003); (2) 60–75 min—metabolism and synthesis of DNA and other macromolecules is in progress, and as revealed by microscopy, replicated chromosomes have commenced segregation; (3) 90 min—segregation of chromosomes has been completed and another round of DNA replication is in progress; and (4) 120 min—cells are actively growing and dividing. As shown in Figure 2c–e, spores with single mutations progressed through outgrowth and on to chromosome segregation, replication, and cell division at about the same rate as did wild‐type spores. In contrast, spores lacking DisA/UvrA or DisA/Mfd exhibited a delay of around 30 min in initiating outgrowth and completing chromosome replication compared to wild‐type spores (Figure 2f–g; Table 1). A detailed analysis of 200 individual germinated/outgrowing spores revealed that 85–90% of the wild‐type spores and those from the *disA*,* uvrA,* and *mfd* strains exhibited a replicated and segregated chromosome by 90 min after mixing with germination medium. In contrast, only ~60% of the Δ*disA/uvrA* and 18% of the Δ*disA/mfd* spores exhibited replicated and completely segregated chromosomes, at this same time (Table 1). In summary, the microscopic evidence together with results presented in Figure 1, strongly suggest that DisA together with Mfd (TCR) or with UvrA (NER) play a crucial role in repairing spontaneous DNA lesions that interfere with replication and thus delay spore outgrowth. To better support this notion, 1 × 10^8^ dormant spores of the WT or ∆*disA uvrA* and ∆*disA mfd* strains were induced to germinate and the DNA content from the same amount of cells was determined at 30, 60, 90, and 120 min. The results showed that the DNA content 90 and 120 min after mixing spores with germinants, was significantly lower in outgrowing spores of *disA*/*uvrA* and *disA*/*mfd* strains than in outgrowing wild‐type spores (Figure 3).

**Table 1:** Percentage of outgrown spores of different *B. subtilis* strains carrying a replicated and segregated chromosome 90 min after the germination onset

Strain (% of outgrown spores carrying a replicated and segregated chromosome)					
Wild‐type (90)	∆*disA* (90)	∆*uvrA* (85)	∆*mfd* (85)	∆*disA uvrA* (60)	∆*disA mfd* (18)

Spores of wild‐type, *∆disA*,* ∆uvrA*,* ∆mfd*,* ∆disA uvrA,* and *∆disA mfd* strains were germinated and outgrown in 2 × SG medium at 37°C. 90 min after the germination onset, at least 200 outgrown spores of each strain that were stained with DAPI were analyzed by fluorescence microscopy in at least six different fields to determine the number of cells showing replication and segregation of its chromosome.

### 3.3 Hydrogen peroxide exacerbates the outgrowth defect in ∆disA uvrA and ∆disA mfd spores

**Figure 4:**
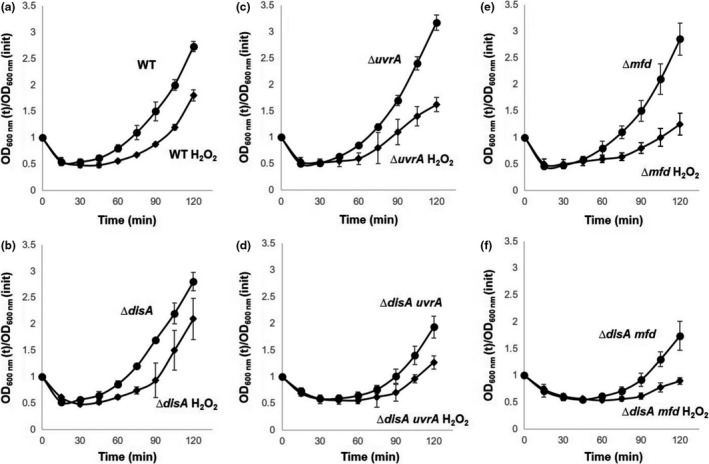
Germination and outgrowth of spores of different *B. subtilis* strains with and without H_2_O_2_. Dormant spores of (a) wild‐type, (b) ∆*disA*, (c) ∆*uvrA*, (d) ∆*disA uvrA,* (e) ∆*mfd, and* (f) ∆*disA mfd* strains were heat shocked and germinated in the absence (●) or presence (◆) of H_2_O_2_. Where indicated, cultures were made 0.5 mmol L^‐1^ in H_2_O_2_ 15 min after initiation of germination. Spore germination and outgrowth were measured by monitoring the OD_600_ of the cultures, as described in Materials and Methods. Values are averages ± standard deviations for triplicate determinations, with different lots of spores; WT, wild type

We next investigated whether oxidative stress is involved in the outgrowth defect exhibited by spores deficient for damage scanning, transcription‐coupled, or excision repair functions. To this end, spores of the wild‐type strain and those bearing single *disA*,* mfd,* or *uvrA* mutations or double mutations in *disA*/*mfd* or *disA*/*uvrA* were challenged with 0.5 mmol L^−1^ H_2_O_2_ 15 min after the initiation of germination, and the effect of the oxidizing agent was monitored by following the OD_600_ nm of cultures. Of note, WT vegetative cells showed an LD_50_ value for H_2_O_2_ treatment of 59.7 ± 4.1 mmol L^−1^; namely, ~120× above the concentration employed in our spore germination/outgrowth experiments. As shown in Figure 4, the outgrowth of spores lacking DisA was slightly delayed by H_2_O_2_, although this effect was not different from that seen with outgrowing wild‐type spores. However, in comparison with wild‐type spores, the outgrowth of ∆*mfd* spores was more affected by H_2_O_2_ addition and this effect was exacerbated in Δ*mfd* spores that also lacked DisA (Figure 4e–f). The delay induced by H_2_O_2_ in spore outgrowth was also evident in UvrA‐deficient spores (Figure 4c), although this effect was only slightly increased in spores of the ∆*disA uvrA* strain (Figure 4D). Taken together, these results strongly suggest that ROS‐induced DNA lesions are involved in the outgrowth defect exhibited by spores lacking *disA*/*mfd* or *disA*/*uvrA*.

### 3.4 DisA and TCR are required during spore outgrowth to contend with the genotoxic effects of 4‐NQO

**Figure 5:**
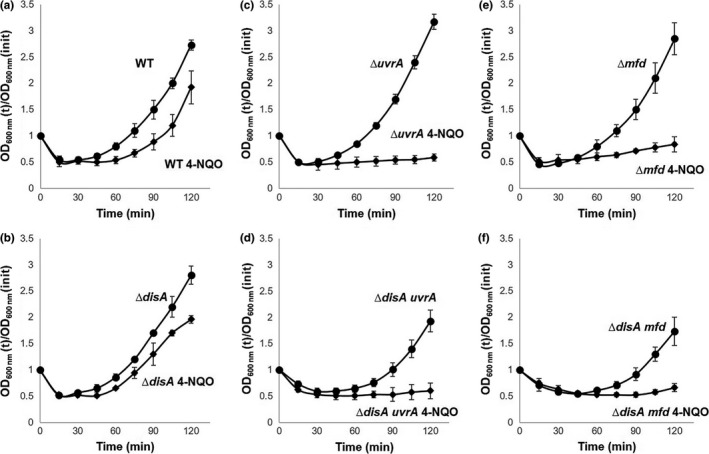
Germination and outgrowth of spores of different *B. subtilis* strains with and without 4‐NQO. Dormant spores of (a) wild‐type, (b) ∆*disA*, (c) ∆*uvrA*, (d) ∆*disA uvrA,* (e) ∆*mfd, and* (f) ∆*disA mfd* strains were heat shocked and germinated in the absence (●) or presence (◆) of 4‐NQO.Where indicated, cultures were made 2 μmol L^‐1^ in 4‐NQO 15 min after the initiation of germination. Spore germination and outgrowth were measured by monitoring the OD_600_ of the cultures, as described in Materials and Methods. Values are averages ± standard deviations for triplicate determinations with different lots of spores; WT, wild type

As noted above, disruption of UvrA or Mfd that play prominent roles in general (NER) and (TCR) excision repair pathways (Truglio, Croteau, Van Houten, & Kisker, 2006) affected the normal progression into vegetative growth in the absence of the checkpoint damage protein DisA. In sporulating cells, the NER and TCR pathways are involved in eliminating lesions from DNA that interfere with replication and transcription (Ramírez‐Guadiana et al., 2013, 2016). To better assess the role of the NER and TCR pathways in spore′s return to life, 15 min after germination was initiated, spores of wild‐type and mutant strains were challenged with 4‐NQO, a genotoxic agent that attacks DNA generating C_8_‐ and N_2_‐guanine and N_6_‐ adenine adducts (Galiègue‐Zouitina, Bailleul, & Loucheux‐Lefebvre, 1985; Tada & Tada, 1976). These DNA lesions have the potential to block replication and transcription in bacteria (Jarosz, Godoy, Delaney, Essigmann, & Walker, 2006) and avian cells (Edmunds, Simpson, & Sale, 2008). In *B. subtilis*, 4‐NQO adducts are mainly eliminated from DNA by the NER and homologous recombination repair pathways (Alonso, Tailor, & Lüder, 1988; Friedman & Yasbin, 1983). Our results revealed that wild‐type and DisA‐deficient spores were only marginally affected by 2 μmol L^−1^ 4‐NQO (Figure 5a,b). In marked contrast, the drug severely slowed the return to vegetative growth of UvrA‐ or Mfd‐deficient spores (Figure 5c,e). A similar effect was caused by 4‐NQO in spores of the Δ*disA uvrA* and Δ*disA mfd* strains (Figure 5d,f). Together, these results support the idea that the global and TCR‐dependent NER repair pathways are both active during outgrowth to deal with lesions that may potentially compromise events such as DNA synthesis and gene expression in this period of development.

### 3.5 DisA modulates mutagenesis in spore outgrowth in coordination with Mfd or UvrA

**Figure 6:**
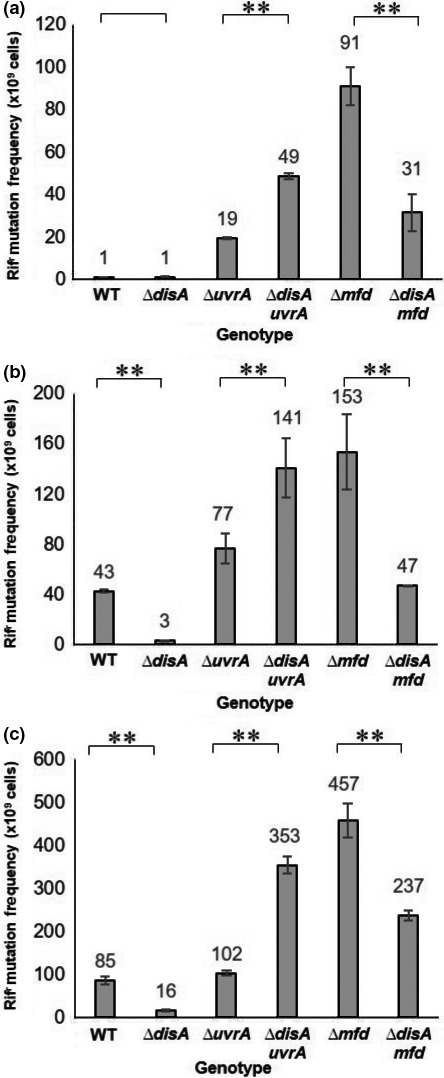
Spontaneous and H_2_O_2_‐ or 4‐NQO‐ induced mutation frequencies of outgrown spores of different *B. subtilis* strains. Dormant spores of different strains were heat shocked and germinated, and 15 min after the initiation of germination cultures were left untreated (a), made 0.5 mmol L^‐1^ in H_2_O_2_ (b) or 2 μmol L^‐1^ in 4‐NQO (c). After 180 min of incubation the Rif^r^ mutation frequency in the cultures was determined as described in Materials ad Methods. Each bar represents the mean of data collected from three independent experiments with different lots of spores, and error bars represent the standard deviation. **, *p *<* *.01 (by the Mann‐Whitney U)

Spores lacking DisA/Mfd or DisA/UvrA displayed a delayed return to vegetative growth, which was exacerbated by H_2_O_2_ or 4‐NQO (Figures 4, 5). As proposed above, the retarded outgrowth of these spores presumably was due to DNA lesions in outgrowing spores which interfered with transcription and replication. To investigate this issue, the spontaneous and induced mutation frequency to rifampicin resistance (Rif^r^) was determined in spores that were induced to germinate and allowed to experience outgrowth and several rounds of cell division for a period of 180 min. As shown in Figure 6a, in comparison with wild‐type spores, the absence of UvrA or Mfd but not of DisA greatly increased levels of Rif^r^ mutants in outgrowing spores. Importantly, the spontaneous mutation frequencies to Rif^r^ of ∆*disA uvrA* and ∆*disA mfd* strains were ~14‐ and 33‐fold lower in exponentially growing vegetative cells than in spores of the same strains undergoing germination/outgrowth (Figs. 6 and S1).

As described above, metabolic conditions prevailing in outgrowing spores promote the synthesis of ROS, which attack DNA and induce the formation of different types of mutagenic lesions (Wang, Kreutzer, & Essigmann, 1998). In support of this notion, the ROS promoting agent, H_2_O_2_ also increased the mutation frequency of outgrowing wild‐type spores and of outgrowing *∆disA*,* ∆mfd*,* ∆uvrA*,* ∆disA*/*mfd,* and *∆disA*/*uvrA* spores (Figure 6b).

We next investigated whether DNA lesions that interfere with DNA replication and potentially affect transcription also contribute to mutagenesis during spore outgrowth. Our results supported this contention, since addition of 4‐NQO during spore germination increased the levels of mutagenesis in outgrowing wild‐type spores and in spores carrying single or double mutations in *disA*/*mfd* and *disA*/*uvrA* (Figure 6c).

Notably, analysis of spontaneous, H_2_O_2_ or 4‐NQO‐induced mutagenesis revealed that disruption of *disA* increased the levels of mutagenesis of UvrA‐deficient spores, whereas the loss of DisA decreased the mutation frequency in ∆*mfd* spores (Figure 6a–c). To investigate if DisA increases the mutagenesis in the absence of Mfd through a pathway involving low‐fidelity DNA polymerases, we disrupted the *yqjH* or *yqjW* genes encoding Y‐family DNA polymerases in the *∆disA/mfd* strain. Notably, the mutation frequency to Rif^r^ in outgrowing ∆*yqjH disA mfd* or ∆*yqjW disA mfd* spores decreased 5‐ or 26‐fold, respectively, compared to that in outgrowing ∆*disA mfd* spores (Figure S2). These results indicate that during spore outgrowth, the absence of Mfd leads to low‐fidelity DisA‐promoted DNA repair.

### 3.6 Base substitutions derived from DNA oxidation and deamination promote mutagenesis during spore outgrowth

**Figure 7:**
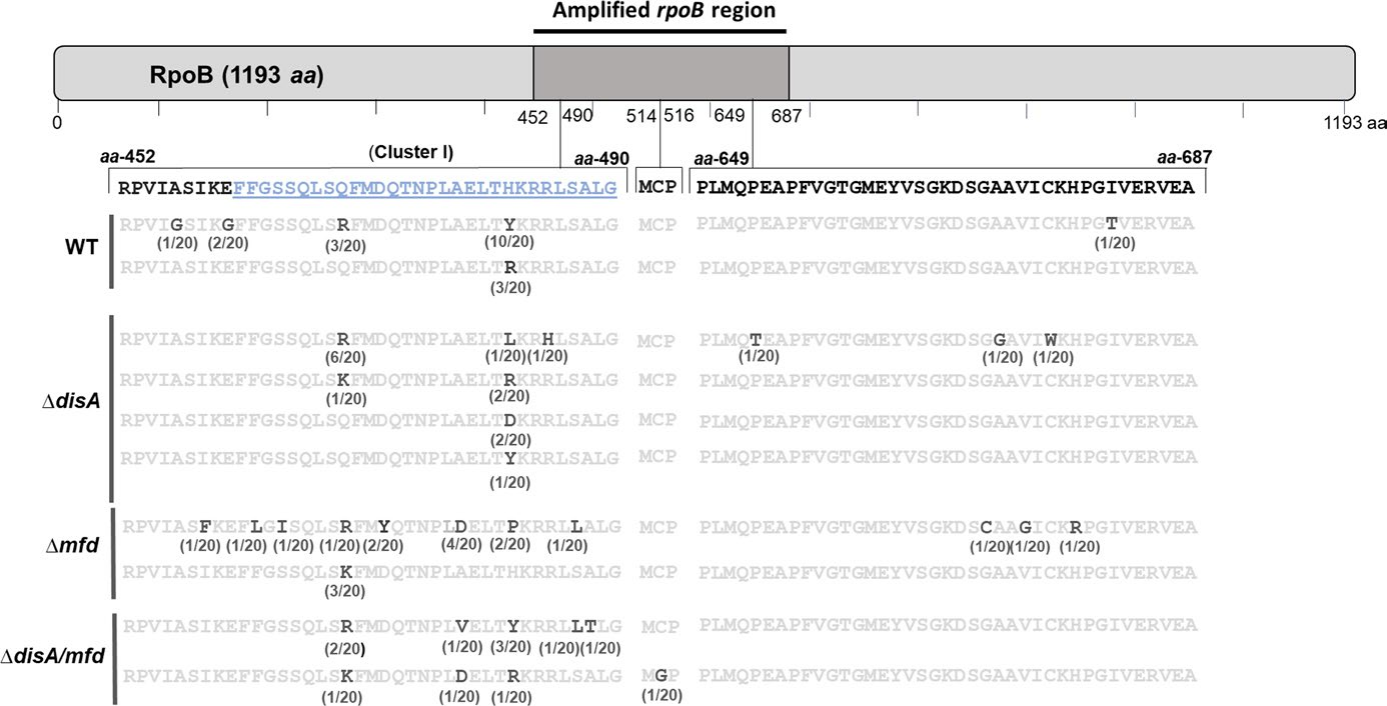
Mutations in clusters I‐III of RNAP β subunit detected in different *B. subtilis* strains. The bar represents the amino acid sequence of the RNAP β subunit (1193 *aa*). The PCR‐amplified and sequenced region (716‐bp from *rpoB*) included the hot‐spot Clusters I‐III associated to Rif^r^ substitutions (Campbell et al., 2001). The position of the Rif^r^ substitutions corresponding to *aa* regions 452‐490, 514‐516 and 649‐687 from RpoB are shown in brackets. The Cluster I in which most of the Rif^r^ mutations were detected is shown in underlined blue bold letters. The WT amino acid sequence for each strain analyzed is shown in light‐gray bold letters and the amino acid change associated with the Rif^r^ phenotype is shown in black bold letters. The frequency of a particular mutation is shown below each amino acid change. *aa* (amino acid)

To further investigate the types of spontaneous mutations occurring in germinated spores after experiencing outgrowth and cell division, a number of Rif^r^ colonies of the wild‐type and various mutant strains were randomly chosen. A 716‐bp fragment from the *rpoB* ORF encompassing the three hot‐spot clusters giving rise to rifampicin resistance in various bacteria (Campbell et al., 2001) was PCR‐amplified from each colony; DNA sequencing confirmed that the Rif^r^ phenotype in all but four of the colonies analyzed was due to base substitutions that occurred in the amplified region of the corresponding *rpoB* gene (Table 2). Interestingly, the major proportion of amino acid changes associated with the Rif^r^ phenotype occurred in the cluster I of the *rpoB* open reading frame (Figure 7) (Campbell et al., 2001). Furthermore, the Rif^r^ mutations identified had a large proportion of A→G and C→T transitions in the outgrowing spores of both the wild‐type and mutant strains (Table 2); these base substitutions are typical mutations produced by deamination of adenine to hypoxanthine or cytosine to uracil (Friedberg, Walker, Siede, & Wood, 2006). The disruption of *disA* promoted the appearance of A↔T transversions, G→A transitions as well as C→G and T→C substitutions, which have been reported to be elicited by oxidative stress (Wang et al., 1998). Interestingly, the A→T, T→G and C→A transversions that were absent in *rpoB* of outgrown wild‐type spores were present in Rif^r^ colonies from Δ*disA* and Δ*mfd* spores (Table 2). However, the base substitutions G→T and A→C that were detected in ∆*mfd* spores were absent in the wild‐type spores. Remarkably, in Rif^r^ colonies derived from outgrowing DisA/Mfd‐deficient spores G→A and G↔T substitutions predominated as well as the C→A transversion detected in outgrown spores of Δ*disA* and Δ*mfd* strains (Table 2). Notably, from 20 Rif^r^ colonies analyzed in the ∆*disA mfd* strain, only 16 exhibited a base substitution in the sequenced *rpoB* region (Table 2). Thus, base substitutions in a different *rpoB* region may have generated the additional 4 colonies with a Rif^r^ phenotype; indeed, mutations occurring in the N‐terminal region of RpoB (from amino acids 132–136) have been reported to produce Rif^r^‐resistant bacteria (Campbell et al., 2001).

**Table 2:** Spectrum of Rif^r^ mutants generated during outgrowth and subsequent cell division of spores of different *B. subtilis* strains

Base substitutions	Rif^r^ strains							
	Wild type		*disA*		*mfd*		*disA mfd*	
	Frequency^a^	Position of mutation^b^	Frequency^a^	Position of mutation^b^	Frequency^a^	Position of mutation^b^	Frequency^a^	Position of mutation^b^
A→T			1/20	(1445)/1	1/20	(1372)/1		
T→A			1/20	(1374)/1				
A→G	8/20	(1379)/2 (1406)/3 (1445)/3	8/20	(1406)/6 (1445)/2	2/20	(1406)/1 (2033)/1	3/20	(1406)/2 (1445)/1
G→A			2/20	(1454)/1 (1956)/1			2/20	(1462)/1 (1959)/1
T→G			1/20	(2028)/1	3/20	(1386)/1 (2013)/1 (2021)/1	1/20	(1543)/1
G→T					4/20	(1391)/1 (1414)/2 (2011)/1	1/20	(1428)/1
G→C								
C→G	1/20	(1367)/1	3/20	(1444)/2 (2015)/1				
T→C	1/20	(2042)/1	1/20	(2040)/1				
C→T	10/20	(1444)/10	1/20	(1444)/1	1/20	(1460)/1	7/20	(1383)/1 (1433)/1 (1444)/3 (1460)/1 (2034)/1
** A→C**					2/20	(1445)/2		
** C→A**			2/20	(1405)/1 (1957)/1	7/20	(1405)/3 (1433)/4	2/20	(1405)/1 (1432)/1

a Frequency→Number of clones with the specific base substitution/number of clones sequenced.

b Position of mutation→Position of mutation in *rpoB*/number of clones with that mutation.

Together, our results suggest that in germinating/outgrowing spores of *B. subtilis*: (1) spontaneous events of base deamination and oxidation contribute to transcriptional and replicative interference; and (2) Mfd and DisA operate on these types of lesions coordinating faithful and error‐prone events of DNA repair.

### 3.7 The SOS response is spontaneously activated during spore germination/outgrowth

**Figure 8:**
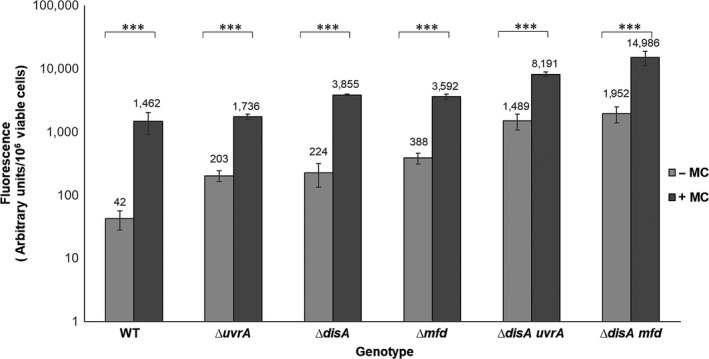
Effect of the lack of Mfd/DisA and UvrA/DisA on the SOS response of *B. subtilis* spores during outgrowth. Spontaneous and induced SOS responses were determined by measuring the expression of a P*recA‐gfpmut3a* fusion. Spores from different *B. subtilis* strains harboring the *precA‐gfpmut3a* fusion, were treated or not with M–C (250 ng ml^‐1^) fifteen min after induction of germination, and 60 min later the fluorescence emission intensities from the cells were quantified. Results presented are average values from three independent experiments, and error bars represent the standard deviation. ***, *p *<* *.001 (by the Mann‐Whitney *U*)

We investigated if the lack of Mfd and UvrA in the Δ*disA* background induces the SOS response during spore germination/outgrowth employing a *recA‐gfpmut3a* fusion that was recombined into the *amyE* locus of the WT and different mutant spores. As shown in Figure 8, outgrowing spores bearing single ∆*uvrA*, ∆*disA*, ∆*mfd*, or double ∆*disA uvrA* and ∆*disA mfd* mutations, respectively, increased ~6 and ~40 times the expression levels of the *gfp3a* fusion in reference to spores of the WT strain. Of note, the RecA‐dependent GFP levels were increased even more in the WT and mutant spores following the addition of the DNA‐damaging agent Mitomycin‐C (Figure 8). Taken together, these results strongly suggest that single‐stranded DNA regions that are increased by spontaneous DNA lesions and replication fork stress can activate the SOS response during spore′s return to vegetative growth.

## 4 DISCUSSION

In this work, an interaction of DisA with Mfd and UvrA (NER) in processing genetic lesions that are potential blocks for transcription and replication during spore outgrowth was uncovered. Thus, wild‐type and *disA*‐deficient spores treated or not with H_2_O_2_ exhibited germination/outgrowth curves and kinetics of chromosomal replication that were essentially similar (Figures 1, 2), suggesting that repair mechanism(s) operating in this developmental stage could suppress the checkpoint function of DisA. Spontaneous DNA lesions reported to stall RNA polymerase and potentially generate transcription‐replication conflicts in different bacteria (Saxowsky & Doetsch, 2006; Wang et al., 1998) were also detected in outgrowing wild‐type spores (Table 2). A prominent role for Mfd in solving these cytotoxic conflicts in replicating cells of *E. coli* and *B. subtilis* has been reported (Million‐Weaver et al., 2015). A further study has also attributed a role for TCR (Mfd/NER) in counteracting the noxious effects of double helix distorter agents during *B. subtilis* sporulation (Ramírez‐Guadiana et al., 2013). Of note, *mfd* and *uvrA* are initially expressed in both sporangial compartments, but only in the forespore in the last stages of sporulation (Ramírez‐Guadiana et al., 2013). A similar pattern of transcription was reported for repair proteins that are packaged in spores and employed to eliminate DNA lesions during spores’ return to vegetative growth (Ayala‐García et al., 2016; Pedraza‐Reyes, Gutiérrez‐Corona, & Nicholson, 1994; Pedraza‐Reyes et al., 2012). Our results showed that, whereas the disruption of *mfd* alone did not affect spore outgrowth or chromosomal replication, such processes were significantly altered after disrupting *mfd* in DisA‐deficient spores. Notably, outgrown spores deficient for either Mfd or DisA exhibited similar patterns of base substitutions (Table 2) suggesting that both proteins operate over the same DNA lesions during spore's return to vegetative growth. Thus, it is possible that in Mfd/DisA‐deficient spores, the RNA polymerase stalled at DNA lesions presents a potential block to transcription, which ultimately impedes efficient DNA replication during spore outgrowth. Collectively these results unveil a novel role for Mfd together with DisA to protect germinating/outgrowing *B. subtilis* spores against spontaneous lesions that potentially compromise transcription and replication. Indeed, ROS‐promoted DNA lesions, including AP sites and 8‐OxoGs have been detected in dormant *B. subtilis* spores (Campos et al., 2014). In connection with these concepts, after the loss of dormancy, spores enter into an active stage of transcription preceding the first round of chromosomal replication (Keijser et al., 2007). Therefore, Mfd could be necessary to couple repair of lesions arresting the progression of the RNA polymerase in the transcribed strand of genes necessary for an efficient spore's return to vegetative growth. However, Mfd could be also involved in resolving structural conflicts resulting from encounters of the replication machinery with RNA polymerase stalled at DNA lesions (Merrikh, Zhang, Grossman, & Wang, 2012; Million‐Weaver et al., 2015). In support of these notions, the outgrowth of spores lacking Mfd or UvrA (NER) was severely affected by 4‐NQO, a DNA‐damaging agent that interferes with DNA replication (Figure 5). Therefore, the TCR and the NER pathways are not only crucial in sporulation (Ramírez‐Guadiana et al., 2013) but as demonstrated here, also during spore outgrowth.

Previous studies have reported on the contribution of UvrA in processing AP sites and single‐strand breaks during spore germination/outgrowth (Campos et al., 2014; Ibarra et al., 2008). Results from this work showed that UvrA (NER) could also back up the function of DisA, as the absence of both proteins affected spore outgrowth in the presence or absence of H_2_O_2_. In parallel with this defect, outgrowing ∆*disA uvrA* spores were delayed in their first chromosomal replication (Figure 2) and exhibited increased spontaneous Rif^r^ mutagenesis (Figure 6). Thus, during spore outgrowth, DisA and UvrA act coordinately to faithfully remove spontaneous genetic lesions that are potential blocks for replication. Of note, the role of UvrA in this developmental stage could be attributed in part to its contribution to TCR.

As discussed above, Mfd and DisA counteract oxidative‐promoted DNA damage during spore outgrowth. Analyses of mutation frequencies to Rif^r^ during spore outgrowth, unveiled an antimutagenic role for Mfd and UvrA. Furthermore, whereas the absence of DisA‐promoted mutagenesis of H_2_O_2_‐treated UvrA‐deficient spores, an opposite effect was observed in outgrown spores of the ∆*mfd* mutant. Of note, genetic disabling of *yqjH* or *yqjW* decreased the mutation frequency even further in outgrown Mfd/DisA‐deficient spores (Figure S2). Together, these results strongly suggest that in spores returning to vegetative growth, spontaneous DNA lesions can be faithfully processed through NER or Mfd as well as in an error‐prone manner through coordination of Mfd with DisA. Previous evidence has associated Mfd with counteracting the noxious effects of bulky lesions during growth and sporulation (Ayora et al., 1996; Ramírez‐Guadiana et al., 2013, 2016) and regulating transcription‐associated mutagenesis (Gómez‐Marroquín et al., 2016; Martin, Pedraza‐Reyes, Yasbin, & Robleto, 2012; Pybus et al., 2010). The evidence presented here also reveals a role for Mfd in coordinating with DisA to process ROS‐promoted lesions of DNA spontaneously generated by the metabolic conditions operating in germinating/outgrowing spores. Our new data also suggest that these oxidative lesions could be responsible for conflicts in replication‐transcription. ROS directly or indirectly attack DNA generating a myriad of DNA lesions, including the oxidative products 8‐OxoG, 8‐OxoA, 2‐OxoA, 5‐OxoC, and thymine glycol (Tg), as well as products of guanine, adenine and cytosine deamination including xanthine, hypoxanthine, and uracil, respectively, among others (Chernikov, Gudkov, Shtarkman, & Bruskov, 2007; Cooke, Evans, Dizdaroglu, & Lunec, 2003). Although these lesions are mainly subjected to BER in an error‐free manner (Dalhus, Laerdahl, Backe, & Bjørås, 2009), alternative modes of error‐prone repair involving Mfd and excision repair proteins have been postulated in bacteria (Brégeon, Doddridge, You, Weiss, & Doetsch, 2003; Gómez‐Marroquín et al., 2016; Million‐Weaver et al., 2015; Wimberly, Chandan Shee, Thornton, Rosenberg, & Hastings, 2014). To further explore mechanistic aspects of repair events during spore outgrowth, we sequenced the *rpoB* gene from spontaneous Rif^r^ colonies of outgrown spores proficient or deficient for *disA*,* mfd,* and *disA/mfd*. Results revealed that A→G and C→T transitions resulting from adenine and cytosine deamination predominated in wild‐type and mutant outgrown spores. Interestingly, DisA‐ and Mfd‐deficient outgrown spores displayed similar mutational spectra, which in addition to the substitutions detected in WT spores, included A→T and T→G transversions promoted by the oxidized bases 2‐OxoA and by unrepaired oxidation product of thymine, 5‐FOdU (Wang et al., 1998). Furthermore, the C→A transversion, which is commonly generated by unrepaired 8‐OxoG (Wang et al., 1998) was also detected in outgrown spores lacking DisA or Mfd. Importantly, G→T and A→C transversions resulting from incorporation of 8‐oxoG into DNA as well as G→A mutations promoted by 5‐OxoC (Wang et al., 1998), were identified in outgrown spores lacking Mfd or DisA, respectively. In conjunction, these results support the notion that DisA and Mfd work together to eliminate ROS‐promoted nonbulky DNA lesions during spore outgrowth, suggesting a role of Mfd and DisA in coordinating proteins involved in repairing these lesions. Notably, 10 out of the Rif^r^ colonies generated from 20 outgrown WT spores that were allowed to progress to the growth stage consisted of the same C→T transition, changing codon H_482_ (CAC) to Y_482_ (TAC) (Table 2, Figure 7). Importantly, this mutation was found to confer rifampicin resistance in *B. subtilis* spores (Nicholson & Maughan, 2002). Furthermore, 3 of the 20 Rif^r^ colonies contained an A→G substitution, which changed codon H_482_ (CAC) to R_482_ (CGC) and this mutation was found not only in spores but also in vegetative cells of *B. subtilis* (Nicholson & Maughan, 2002). Therefore, physiological conditions encountered by outgrowing *B. subtilis* spores not only potentiate mutagenesis but also generate a differential mutational spectra with respect to that exhibited by vegetative cells. Finally, the A↔T, G↔C, T→C and A→C mutations were absent in outgrown *∆disA/mfd* spores, strongly suggesting that additional repair function(s) eliminate 2‐OxoA, 5‐OxoC, and 8‐OxoG from the outgrowing spore's chromosome.

Importantly, additional experiments revealed that spontaneous DNA lesions or their repair intermediates detected in outgrown spores of the WT and repair‐deficient strains activated the expression of a *recA‐gfp* fusion (Figure 8). These results revealed that the SOS response is active in outgrowing *B. subtilis* spores, in addition to the roles of DisA, Mfd and UvrA in repair of ROS‐promoted DNA damage during this developmental stage. On the basis of these observations, we postulate that repair intermediaries of these oxidative lesions can stall RNA polymerase, the absence of Mfd precludes dislodging the stalled polymerase from DNA, and this eventually stalls the DNA replication machinery as well. In agreement with this notion, it has been proposed that a MutY‐AP site complex stalls RNA polymerase during transcription of the mutant *leuC427* gene and activates an Mfd‐dependent event leading to leucine prototrophy (Gómez‐Marroquín et al., 2016). On the other hand, the DisA‐dependent error‐prone repair pathway could be activated by other DNA structures, including branched DNA as well as stalled replication forks (Gándara et al., 2017; Witte et al., 2008). In support of this hypothesis, a recent report revealed the involvement of PolY1 (YqjH) and PolY2 (YqjW) in modulating mutagenic events in DisA‐deficient growing *B. subtilis* cells (Raguse et al., 2017). Overall, the results described in this study provide novel information regarding the interactive function of Mfd and DisA to dynamically counteract the cytotoxic and genotoxic effects of ROS‐promoted lesions during the return to life of *B. subtilis* spores.

## CONFLICT OF INTEREST

None declared.

## References

[mbo3593-bib-0001] Alonso, J. C. , Tailor, R. H. , & Lüder, G. (1988). Characterization of recombination‐deficient mutants of *Bacillus subtilis* . Journal of Bacteriology, 170, 3001–3007. 10.1128/jb.170.7.3001-3007.1988 3133357 PMC211241

[mbo3593-bib-0002] Ayala‐García, V. M. , Valenzuela‐García, L. I. , Setlow, P. , & Pedraza‐Reyes, M. (2016). Aag hypoxanthine‐DNA glycosylase is synthesized in the forespore compartment and involved in counteracting the genotoxic and mutagenic effects of hypoxanthine and alkylated bases in DNA during *Bacillus subtilis* sporulation. Journal of Bacteriology, 198, 3345–3354. 10.1128/JB.00625-16 27698084 PMC5116938

[mbo3593-bib-0003] Ayora, S. , Rojo, F. , Ogasawara, N. , Nakai, S. , & Alonso, J. C. (1996). The Mfd protein of *Bacillus subtilis* 168 is involved in both transcription‐coupled DNA repair and DNA recombination. Journal of Molecular Biology, 256, 301–318. 10.1006/jmbi.1996.0087 8594198

[mbo3593-bib-0004] Bagyan, I. , Casillas‐Martinez, L. , & Setlow, P. (1998). The *katX* gene, which codes for the catalase in spores of *Bacillus subtilis*, is a forespore‐specific gene controlled by σ^F^, and KatX is essential for hydrogen peroxide resistance of the germinating spore. Journal of Bacteriology, 180, 2057–2062. 9555886 10.1128/jb.180.8.2057-2062.1998 PMC107130

[mbo3593-bib-0005] Barajas‐Ornelas, R. C. , Ramírez‐Guadiana, F. H. , Juárez‐Godínez, R. , Ayala‐García, V. M. , Robleto, E. A. , Yasbin, R. E. , & Pedraza‐Reyes, M. (2014). Error‐prone processing of apurinic/apyrimidinic (AP) sites by PolX underlies a novel mechanism that promotes adaptive mutagenesis in *Bacillus subtilis* . Journal of Bacteriology, 196, 3012–3022. 10.1128/JB.01681-14 24914186 PMC4135629

[mbo3593-bib-0006] Bejerano‐Sagie, M. , Oppenheimer‐Shaanan, Y. , Berlatzky, I. , Rouvinski, A. , Meyerovich, M. , & Ben‐Yehuda, S. (2006). A checkpoint protein that scans the chromosome for damage at the start of sporulation in *Bacillus subtilis* . Cell, 125, 679–690. 10.1016/j.cell.2006.03.039 16713562

[mbo3593-bib-0007] Brégeon, D. , Doddridge, Z. A. , You, H. J. , Weiss, B. , & Doetsch, P. W. (2003). Transcriptional mutagenesis induced by uracil and 8‐oxoguanine in *Escherichia coli* . Molecular Cell, 12, 959–970. 10.1016/S1097-2765(03)00360-5 14580346

[mbo3593-bib-0008] Burkholder, W. F. , Kurtser, I. , & Grossman, A. D. (2001). Replication initiation proteins regulate a developmental checkpoint in *Bacillus subtilis* . Cell, 104, 269–279. 10.1016/S0092-8674(01)00211-2 11207367

[mbo3593-bib-0009] Campbell, E. A. , Korzheva, N. , Mustaev, A. , Murakami, K. , Nair, S. , Goldfarb, A. , & Darst, S. A. (2001). Structural mechanism for rifampicin inhibition of bacterial RNA polymerase. Cell, 104, 901–912. 10.1016/S0092-8674(01)00286-0 11290327

[mbo3593-bib-0010] Campos, S. S. , Ibarra‐Rodriguez, J. R. , Barajas‐Ornelas, R. C. , Ramírez‐Guadiana, F. H. , Obregón‐Herrera, A. , Setlow, P. , & Pedraza‐Reyes, M. (2014). Interaction of apurinic/apyrimidinic endonucleases Nfo and ExoA with the DNA integrity scanning protein DisA in the processing of oxidative DNA damage during *Bacillus subtilis* spore outgrowth. Journal of Bacteriology, 196, 568–578. 10.1128/JB.01259-13 24244006 PMC3911150

[mbo3593-bib-0011] Chernikov, A. V. , Gudkov, S. V. , Shtarkman, I. N. , & Bruskov, V. I. (2007). Oxygen effect in heat‐induced DNA damage. Biophysics, 52, 185–190. 10.1134/S0006350907020078 17477051

[mbo3593-bib-0012] Cooke, M. S. , Evans, M. D. , Dizdaroglu, M. , & Lunec, J. (2003). Oxidative DNA damage: Mechanisms, mutation, and disease. The FASEB Journal, 17, 1195–1214. 10.1096/fj.02-0752rev 12832285

[mbo3593-bib-0013] Dalhus, B. , Laerdahl, J. K. , Backe, P. H. , & Bjørås, M. (2009). DNA base repair–recognition and initiation of catalysis. FEMS Microbiology Reviews, 33, 1044–1078. 10.1111/j.1574-6976.2009.00188.x 19659577

[mbo3593-bib-0014] Edmunds, C. E. , Simpson, L. J. , & Sale, J. E. (2008). PCNA ubiquitination and REV1 define temporally distinct mechanisms for controlling translesion synthesis in the avian cell line DT40. Molecular Cell, 30, 519–529. 10.1016/j.molcel.2008.03.024 18498753

[mbo3593-bib-0016] Friedberg, E. C. , Walker, G. C. , Siede, W. , & Wood, R. D. . (Eds.). (2006). DNA repair and mutagenesis. Washington DC: American Society for Microbiology Press.

[mbo3593-bib-0017] Friedman, B. M. , & Yasbin, R. E. (1983). The genetics and specificity of the constutive excision repair system of *Bacillus subtilis* . Molecular and General Genetics, 190, 481–486. 10.1007/BF00331080 6410154

[mbo3593-bib-0018] Galiègue‐Zouitina, S. , Bailleul, B. , & Loucheux‐Lefebvre, M. H. (1985). Adducts from in vivo action of the carcinogen 4‐hydroxyaminoquinoline 1‐oxide in rats and from in vitro reaction of 4‐acetoxyaminoquinoline 1‐oxide with DNA and polynucleotides. Cancer Research, 45, 520–525. 3917848

[mbo3593-bib-0019] Gándara, C. , & Alonso, J. C. (2015). DisA and c‐di‐AMP act at the intersection between DNA‐damage response and stress homeostasis in exponentially growing *Bacillus subtilis* cells. DNA Repair, 27, 1–8. 10.1016/j.dnarep.2014.12.007 25616256

[mbo3593-bib-0020] Gándara, C. , de Lucena, D. K. , Torres, R. , Serrano, E. , Altenburger, S. , Graumann, P. L. , & Alonso, J. C. (2017). Activity and in vivo dynamics of *Bacillus subtilis* DisA are affected by RadA/Sms and by Holliday junction‐processing proteins. DNA Repair, 55, 17–30. 10.1016/j.dnarep.2017.05.002 28511132

[mbo3593-bib-0021] Gómez‐Marroquín, M. , Martin, H. , Pepper, A. , Girard, M. , Kidman, A. , Vallin, C. , Yasbin, R. , Pedraza‐Reyes, M. , & Robleto, E. (2016). Stationary‐phase mutagenesis in stressed *Bacillus subtilis* cells operates by Mfd‐dependent mutagenic pathways. Genes, 7, 33 10.3390/genes7070033 PMC4962003 27399782

[mbo3593-bib-0022] Hanawalt, P. C. , & Spivak, G. (2008). Transcription‐coupled DNA repair: Two decades of progress and surprises. Nature Reviews Molecular Cell Biology, 9, 958–970. 10.1038/nrm2549 19023283

[mbo3593-bib-0023] Ibarra, J. R. , Orozco, A. D. , Rojas, J. A. , López, K. , Setlow, P. , Yasbin, R. E. , & Pedraza‐Reyes, M. (2008). Role of the Nfo and ExoA apurinic/apyrimidinic endonucleases in repair of DNA damage during outgrowth of *Bacillus subtilis* spores. Journal of Bacteriology, 190, 2031–2038. 10.1128/JB.01625-07 18203828 PMC2258865

[mbo3593-bib-0024] Jarosz, D. F. , Godoy, V. G. , Delaney, J. C. , Essigmann, J. M. , & Walker, G. C. (2006). A single amino acid governs enhanced activity of DinB DNA polymerases on damaged templates. Nature, 439, 225–228. 10.1038/nature04318 16407906

[mbo3593-bib-0025] Keijser, B. J. , Ter Beek, A. , Rauwerda, H. , Schuren, F. , Montijn, R. , van der Spek, H. , & Brul, S. (2007). Analysis of temporal gene expression during *Bacillus subtilis* spore germination and outgrowth. Journal of Bacteriology, 189, 3624–3634. 10.1128/JB.01736-06 17322312 PMC1855883

[mbo3593-bib-0026] Martin, H. A. , Pedraza‐Reyes, M. , Yasbin, R. E. , & Robleto, E. A. (2012). Transcriptional de‐repression and Mfd are mutagenic in stressed *Bacillus subtilis* cells. Journal of Molecular Microbiology and Biotechnology, 21, 45–58. 10.1159/000332751 PMC3697266 22248542

[mbo3593-bib-0027] Merrikh, H. , Zhang, Y. , Grossman, A. D. , & Wang, J. D. (2012). Replication–transcription conflicts in bacteria. Nature Reviews Microbiology, 10, 449–458. 22669220 10.1038/nrmicro2800 PMC3467967

[mbo3593-bib-0028] Miller, J. H. (1972). Experiments in molecular genetics. Cold Spring Harbor, NY: Cold Spring Harbor Laboratory Press.

[mbo3593-bib-0029] Million‐Weaver, S. , Samadpour, Ariana. N. , Moreno‐Habel, Daniela. A. , Nugent, P. , Brittnacher, Mitchell. J. , Weiss, E. , Hayden, Hillary. S. , Miller, Samuel. I. , Liachko, I. , & Merrikh, H. (2015). An underlying mechanism for the increased mutagenesis of lagging‐strand genes in *Bacillus subtilis* . Proceedings of the National Academy of Sciences of the United States of America, 112, E1096–E1105. 10.1073/pnas.1416651112 25713353 PMC4364195

[mbo3593-bib-0030] Nicholson, W. L. , & Maughan, H. (2002). The spectrum of spontaneous rifampin resistance mutations in the *rpoB* gene of *Bacillus subtilis* 168 spores differs from that of vegetative cells and resembles that of *Mycobacterium tuberculosis* . Journal of Bacteriology, 184, 4936–4940. 10.1128/JB.184.17.4936-4940.2002 12169622 PMC135274

[mbo3593-bib-0031] Nicholson, W. L. , Munakata, N. , Horneck, G. , Melosh, H. J. , & Setlow, P. (2000). Resistance of *Bacillus* endospores to extreme terrestrial and extraterrestrial environments. Microbiology and Molecular Biology Reviews, 64, 548–572. 10.1128/MMBR.64.3.548-572.2000 10974126 PMC99004

[mbo3593-bib-0032] Nicholson, W. L. , & Setlow, P. (1990). Spore, germination and outgrowth In Harwood C. S., & Cutting S. M. (Eds.), Molecular Biological Methods for Bacillus (pp. 391–450). New York: John Wiley & Sons.

[mbo3593-bib-0033] Oppenheimer‐Shaanan, Y. , Wexselblatt, E. , Katzhendler, J. , Yavin, E. , & Ben‐Yehuda, S. (2011). c‐di‐AMP reports DNA integrity during sporulation in Bacillus subtilis. EMBO Reports, 12, 594–601. 10.1038/embor.2011.77 21566650 PMC3128283

[mbo3593-bib-0034] Paidhungat, M. , & Setlow, P. (2002). Spore germination and outgrowth In Sonenshein A., Losick R., & Hoch J. (Eds.), Bacillus subtilis and its relatives: From genes to cells (pp. 537–548). Washington, D.C.: American Society for Microbiology 10.1128/9781555817992

[mbo3593-bib-0035] Pedraza‐Reyes, M. , Gutiérrez‐Corona, F. , & Nicholson, W. L. (1994). Temporal regulation and forespore‐specific expression of the spore photoproduct lyase gene by sigma‐G RNA polymerase during *Bacillus subtilis* sporulation. Journal of Bacteriology, 176, 3983–3991. 10.1128/jb.176.13.3983-3991.1994 8021181 PMC205596

[mbo3593-bib-0036] Pedraza‐Reyes, M. , Ramírez‐Ramírez, N. , Vidales‐Rodríguez, L. E. , & Robleto, E. A. (2012). Mechanisms of bacterial spore survival In Abel‐Santos E. (Ed.), Bacterial Spores: current Research and Applications (pp. 73–84). Norfolk, U.K.: Caister Academic Press.

[mbo3593-bib-0037] Pybus, C. , Pedraza‐Reyes, M. , Ross, C. A. , Martin, H. , Ona, K. , Yasbin, R. E. , & Robleto, E. (2010). Transcription associated mutation in *Bacillus subtilis* cells under stress. Journal of Bacteriology, 192, 3321–3328. 10.1128/JB.00354-10 20435731 PMC2897666

[mbo3593-bib-0038] Raguse, M. , Torres, R. , Seco, E. M. , Gándara, C. , Ayora, S. , Moeller, R. , & Alonso, J. C. (2017). *Bacillus subtilis* DisA helps to circumvent replicative stress during spore revival. DNA Repair, 59, 57–68. 10.1016/j.dnarep.2017.09.006 28961460

[mbo3593-bib-0039] Rahn‐Lee, L. , Gorbatyuk, B. , Skovgaard, O. , & Losick, R. (2009). The conserved sporulation protein YneE inhibits DNA replication in *Bacillus subtilis* . Journal of Bacteriology, 191, 3736–3739. 10.1128/JB.00216-09 19329632 PMC2681902

[mbo3593-bib-0041] Ramírez‐Guadiana, F. H. , Barajas‐Ornelas, R. C. , Ayala‐García, V. M. , Yasbin, R. E. , Robleto, E. , & Pedraza‐Reyes, M. (2013). Transcriptional coupling of DNA repair in sporulating *Bacillus subtilis* cells. Molecular Microbiology, 90, 1088–1099. 10.1111/mmi.12417 24118570

[mbo3593-bib-0042] Ramírez‐Guadiana, F. H. , Barajas‐Ornelas, R. C. , Corona‐Bautista, S. U. , Setlow, P. , & Pedraza‐Reyes, M. (2016). The RecA‐dependent SOS response is active and required for processing of DNA damage during *Bacillus subtilis* sporulation. PLoS ONE, 11, e0150348 10.1371/journal.pone.0150348 26930481 PMC4773242

[mbo3593-bib-0043] Ramírez‐Guadiana, F. H. , Barraza‐Salas, M. , Ramírez‐Ramírez, N. , Ortiz‐Cortés, M. , Setlow, P. , & Pedraza‐Reyes, M. (2012). Alternative excision repair of ultraviolet B‐and C‐induced DNA damage in dormant and developing spores of *Bacillus subtilis* . Journal of Bacteriology, 194, 6096–6104. 10.1128/JB.01340-12 22961846 PMC3486385

[mbo3593-bib-0044] Ross, C. , Pybus, C. , Pedraza‐Reyes, M. , Sung, H. M. , Yasbin, R. E. , & Robleto, E. (2006). Novel role of *mfd*: Effects on stationary‐phase mutagenesis in *Bacillus subtilis* . Journal of Bacteriology, 188, 7512–7520. 10.1128/JB.00980-06 16950921 PMC1636285

[mbo3593-bib-0045] Sambrook, J. , & Russell, D. W. (2001). Molecular Cloning: A Laboratory Manual, 3rd ed. Cold Spring Harbor, NY: Cold Spring Harbor Laboratory Press.

[mbo3593-bib-0046] Saxowsky, T. T. , & Doetsch, P. W. (2006). RNA polymerase encounters with DNA damage: Transcription‐coupled repair or transcriptional mutagenesis? Chemical Reviews, 106, 474–488. 10.1021/cr040466q 16464015

[mbo3593-bib-0047] Schaeffer, P. , Millet, J. , & Aubert, J. P. (1965). Catabolic repression of bacterial sporulation. Proceedings of the National Academy of Sciences, 54, 704–711. 10.1073/pnas.54.3.704 PMC219731 4956288

[mbo3593-bib-0048] Selby, C. P. , & Sancar, A. (1993). Molecular mechanism of transcription‐repair coupling. Science, 260, 53–58. 10.1126/science.8465200 8465200

[mbo3593-bib-0049] Selby, C. P. , Witkin, E. M. , & Sancar, A. (1991). *Escherichia coli mfd* mutant deficient in” mutation frequency decline” lacks strand‐specific repair: In vitro complementation with purified coupling factor. Proceedings of the National Academy of Sciences, 88, 11574–11578. 10.1073/pnas.88.24.11574 PMC53178 1763073

[mbo3593-bib-0501] Setlow, P. (1975). Protein metabolism during germination of Bacillus megaterium spores. II. Degradation of pre‐existing and newly synthesized proteins. Journal of Biological Chemistry, 250(2), 631–637. 803495

[mbo3593-bib-0050] Setlow, P. (1988). Small, acid‐soluble spore proteins of *Bacillus* species: Structure, synthesis, genetics, function, and degradation. Annual Review of Microbiology, 42, 319–338. 10.1146/annurev.mi.42.100188.001535 3059997

[mbo3593-bib-0051] Setlow, P. (2003). Spore germination. Current Opinion in Microbiology, 6, 550–556. 10.1016/j.mib.2003.10.001 14662349

[mbo3593-bib-0052] Setlow, P. (2007). I will survive: DNA protection in bacterial spores. Trends in Microbiology, 15, 172–180. 10.1016/j.tim.2007.02.004 17336071

[mbo3593-bib-0053] Setlow, P. , & Primus, G. (1975). Protein metabolism during germination of *Bacillus megaterium* spores. I. Protein synthesis and amino acid metabolism. Journal of Biological Chemistry, 250, 623–630. 803494

[mbo3593-bib-0054] Setlow, B. , & Setlow, P. (1996). Role of DNA repair in *Bacillus subtilis* spore resistance. Journal of Bacteriology, 178, 3486–3495. 10.1128/jb.178.12.3486-3495.1996 8655545 PMC178117

[mbo3593-bib-0055] Setlow, P. , Wang, S. , & Li, Y. Q. (2017). Germination of spores of the orders *Bacillales* and *Clostridiales* . Annual Review of Microbiology, 8, 459–477. 10.1146/annurev-micro-090816-093558 28697670

[mbo3593-bib-0056] Tada, M. , & Tada, M. (1976). Main binding sites of the carcinogen, 4‐nitroquinoline 1‐oxide in nucleic acids. Biochimica et Biophysica Acta, 454, 558–566. 10.1016/0005-2787(76)90281-1 826278

[mbo3593-bib-0057] Truglio, J. J. , Croteau, D. L. , Van Houten, B. , & Kisker, C. (2006). Prokaryotic nucleotide excision repair: The UvrABC system. Chemical Reviews, 106, 233–252. 10.1021/cr040471u 16464004

[mbo3593-bib-0058] Wang, D. , Kreutzer, D. A. , & Essigmann, J. M. (1998). Mutagenicity and repair of oxidative DNA damage: Insights from studies using defined lesions. Mutation Research, 400, 99–115. 10.1016/S0027-5107(98)00066-9 9685598

[mbo3593-bib-0059] Wimberly, H. , Chandan Shee, P. C. , Thornton, P. S. , Rosenberg, Susan. M. , & Hastings, P. J. (2014). Corrigendum R loops nicks initiate DNA breakage genome instability in non growing Escherichia coli. Nature Communications, 5, 2115. 10.1038/ncomms3115 PMC3715873 23828459

[mbo3593-bib-0060] Witte, G. , Hartung, S. , Büttner, K. , & Hopfner, K. P. (2008). Structural biochemistry of a bacterial checkpoint protein reveals diadenylate cyclase activity regulated by DNA recombination intermediates. Molecular Cell, 30, 167–178. 10.1016/j.molcel.2008.02.020 18439896

[mbo3593-bib-0061] Zalieckas, J. M. , Wray, L. V. Jr , Ferson, A. E. , & Fisher, S. H. (1998). Transcription‐repair coupling factor is involved in carbon catabolite repression of the *Bacillus subtilis hut* and *gnt* operons. Molecular Microbiology, 27, 1031–1038. 10.1046/j.1365-2958.1998.00751.x 9535092

