# Supplementary material for: Transcriptional coupling (Mfd) and DNA damage scanning (DisA) coordinate excision repair events for efficient Bacillus subtilis spore outgrowth
Source: Microbiologyopen. 2018 Mar 13;7(5):e00593. doi: 10.1002/mbo3.593 (PMC6182552; doi:10.1002/mbo3.593)
Supplement: Supplementary file 1 [file MBO3-7-e00593-s001.docx]

**Title:** Transcriptional coupling (Mfd) and DNA damage scanning (DisA) coordinate excision repair events for efficient *Bacillus subtilis* spore outgrowth

**Authors:** Luz I.Valenzuela-García^a^, Víctor M. Ayala-García^a^, Ana G. Regalado-García^a^, Peter Setlow^b^ and Mario Pedraza-Reyes^a,^ *

**Author’s affiliation:** Department of Biology, University of Guanajuato. 36050 Noria Alta. Guanajuato, Gto. MEXICO^a^. Department of Molecular Biology and Biophysics, UConn Health, Farmington, Connecticut, USA^b^

**Running title:** Mfd/DisA coupling of DNA repair and mutagenesis

*For correspondence. E-mail: pedrama@ugto.mx; Tel. (+52) 473 732 2 00 06, ext. 8161; Fax (+52) 473 73 2 00 06, ext 8153.

**Supporting information**

**Supporting Table S1.** *B. subtilis* strains and plasmids used in this study

| **Strain or plasmid** | **Genotype or description^a^** | **Reference or source^b^** |
| --- | --- | --- |
| **Strains** |  |  |
| 168 | Wild-type; *trpC2* | Laboratory stock |
| PS832 | Wild-type *trpC2* revertant of strain 168 | Laboratory stock |
| PERM733 | *∆disA::lacZ* Er^r^ | Campos *et al*., 2014 |
| PERM938 | *∆mfd::tet* Tc^r^ | Ramírez-Guadiana *et al*., 2013 |
| PERM939 | *∆mfd::tc ∆yqjH::erm* Tc^r^ Er^r^ | Ramírez-Guadiana *et al*., 2013 |
| PERM940 | *∆mfd::tc ∆yqjW::erm* Tc^r^ Er^r^ | Ramírez-Guadiana *et al*., 2013 |
| PERM985 | *∆uvrA::cm* Cm^r^ | Ramírez-Guadiana *et al*., 2012 |
| PERM1135 | *∆mfd*::*tet amyE::empty* Tet^r^ Spc^r^ | Ramírez-Guadiana *et al* 2013 |
| PERM1333 | *∆disA::erm ∆mfd::tc* Er^r^ Tc^r^ | PERM938 →PERM733 |
| PERM1342 | *∆disA::erm ∆uvrA::cm* Er^r^ Cm^r^ | PERM985 →PERM733 |
| PERM1460 | *∆mfd::tet* Tc^r^ | PERM938 →PS832 |
| PERM1461 | *∆uvrA::cm* Cm*^r^* | PERM985 →PS832 |
| PERM1504 | *∆disA::lacZ* Cm^r^ | pPERM1372→PS832 |
| PERM1510 | *∆disA::cm ∆mfd::tet ∆yqjH::erm* Cm^r^ Tc^r^ Er^r^ | PERM939→PERM1504 |
| PERM1511 | *∆disA::cm ∆mfd::tet ∆yqjW::erm* Cm^r^ Tc^r^ Er^r^ | PERM940→PERM1504 |
| PERM1529 | *∆uvrA*::*cm amyE::empty* Cm^r^Spc^r^ | pDR111 →PERM1461 |
| PERM1530 | *∆uvrA*::*cm ∆disA::erm amyE::empty* Cm^r^ Er^r^ Spc^r^ | pDR111 →PERM1342 |
| PERM1548 | *∆uvrA*::*cm amyE*::P_recA_-*gfpmut3a* Cm^r^ Spc^r^ | pPERM1237→PERM1461 |
| PERM1549 | Wild-type; *trpC2* *amyE*::P_recA_-*gfpmut3a* Spc^r^ | pPERM1237→PS832 |
| PERM1550 | *∆disA::erm amyE*::P*_recA_*-*gfpmut3a* Er^r^ Spc^r^ | pPERM1237→PERM733 |
| PERM1559 | *∆uvrA*::*cam ∆disA::erm amyE*::P*_recA_*-*gfpmut3a* Cm^r^ Er^r^ Spc^r^ | pPERM1237→PERM1342 |
| PERM1560 | *∆mfd*::*tet* *∆disA::erm amyE*::P*_recA_*-*gfpmut3a* Tet^r^ Er^r^ Spc^r^ | pPERM1237→PERM1333 |
| PERM1561 | *∆mfd*::*tet amyE*::P*_recA_*-*gfpmut3a* Tc^r^ Spc^r^ | pPERM1237→PERM1460 |
| PERM1570 | Wild type*; trpC2 amyE::empty* Spc^r^ | pDR111 →PS832 |
| PERM1571 | *∆disA::erm amyE::empty* Er^r^ Spc^r^ | pDR111 →PERM733 |
| PERM1646 | *∆mfd*::*tet* *∆disA::erm amyE::empty* Tc^r^ Er^r^ Spc^r^ | pDR111 →PERM1333 |
| **Plasmids** |  |  |
| pMutin4-cat | Integrational *lacZ* fusion vector; Cm^r^ | Barajas-Ornelas *et al*., 2014 |
| pDR111 | *amyE*::Phyper-spank promoter (P*_hs_*) Amp^r^ Spc^r^ | David Rudner |
| pPERM1237 | pDR111 containing the P*_recA_*-*gfpmut3a* construct Amp^r^ Spc^r^ | Ramírez-Guadiana *et al*, 2016 |
| pPERM1372 | pMutin4cat containing an internal region (307 bp) of *disA*; Cm^r^ | This study |

^a^ Selection markers: Er, erythromycin; Cm, chloramphenicol; Tc, Tetracycline; Spc, Spectinomycin.

^b^ X→Y indicates that strain Y was transformed with DNA source X.

**Supporting Materials and Methods**

**Analysis of spontaneous mutation frequencies in growing cells**

For determination of spontaneous mutation frequencies in growing cells, overnight cultures of each strain were inoculated into flasks containing LB medium, grown to OD_600nm_ of 0.5 at 37°C and 180 min after, 10 ml of cell samples were collected, washed with 10 ml of PBS and resuspended in 1 mL of the same buffer. Aliquots of cells were plated on six LB medium plates containing 10 μg ml^-1^ of rifampicin, and Rif^r^ colonies were counted after 2 days of incubation at 37°C. The number of cells used to calculate the frequency of mutation to Rif^r^ was determined by plating aliquots of appropriate dilutions on LB medium plates without rifampicin and incubating the plates for 24 h at 37°C. These experiments were performed in triplicate.

**Supporting References**

Barajas-Ornelas, R.C., Ramírez-Guadiana, F. H., Juárez-Godínez, R., Ayala-García, V. M., Robleto, E. A., Yasbin, R. E., and Pedraza-Reyes, M. (2014). Error-prone processing of apurinic/apyrimidinic (AP) sites by PolX underlies a novel mechanism that promotes adaptive mutagenesis in *Bacillus subtilis*. *J Bacteriol* **196:** 3012-3022.

Campos, S. S., Ibarra-Rodriguez, J. R., Barajas-Ornelas, R. C., Ramírez-Guadiana, F. H., Obregón-Herrera, A., Setlow, P., and Pedraza-Reyes, M. (2014). Interaction of apurinic/apyrimidinic endonucleases Nfo and ExoA with the DNA integrity scanning protein DisA in the processing of oxidative DNA damage during *Bacillus subtilis* spore outgrowth*. J Bacteriol***196:** 568-578.

Ramírez-Guadiana, F. H., Barajas-Ornelas, R.C., Corona-Bautista, S. U., Setlow, P., & Pedraza-Reyes, M. (2016). The RecA-dependent SOS response is active and required for processing of DNA damage during *Bacillus subtilis* sporulation. *PloS one* **11:** e0150348.

Ramírez-Guadiana, F.H., Barajas-Ornelas, R.C., Ayala-García, V.M., Yasbin, R. E., Robleto, E., and Pedraza-Reyes, M. (2013). Transcriptional coupling of DNA repair in sporulating *Bacillus subtilis* cells. *Mol Microbiol* **90:** 1088-1099.

Ramírez-Guadiana, F.H., Barraza-Salas, M., Ramírez-Ramírez, N., Ortiz-Cortés, M., Setlow, P., and Pedraza-Reyes, M. (2012). Alternative excision repair of ultraviolet B-and C-induced DNA damage in dormant and developing spores of *Bacillus subtilis*. *J Bacteriol* **194:** 6096-6104.

**Supporting Figure legends**

**Fig. S1. Spontaneous Rif^r^ mutation frequencies in vegetative cells of differentt *B. subtilis* strains.** Vegetative cells of different strains were grown at 37^o^C/250 rpm in liquid LB medium to an OD_600_ of 0.5. 180 min after this point, spontaneous mutation frequencies were determined in cultures as described in Supporting Materials and Methods. Each bar represents the mean of data collected from three independent experiments with different lots of cells, and error bars represent the standard deviation. ***ns***, non-significant; *, *P*<0.05 (by the Mann-Whitney U test).

**Fig. S2. Spontaneous Rif^r^ mutation frequencies in outgrown spores of differentt *B. subtilis* strains.** Dormant spores of different strains were heat shocked and germinated as described in Experimental procedures. 180 min after initiation of germination, spontaneous mutation frequencies were determined in cultures as described in Materials and Methods. Each bar represents the mean of data collected from three independent experiments with different lots of spores, and error bars represent the standard deviation. **, *P*<0.01 (by the Mann-Whitney U test).

**Valenzuela-Garcia *et al*., Figure S1**

**Valenzuela-Garcia *et al*., Figure S2**
